# Impact of peer-support programs for individuals with autism: A systematic review

**DOI:** 10.1177/13623613251374971

**Published:** 2025-09-16

**Authors:** Monica HM Verkooijen, Marjolijn Ketelaar, Max van Woerden, Wouter G Staal, Indira Tendolkar, Janneke R Zinkstok

**Affiliations:** 1 Radboud University Medical Center, The Netherlands; 2 Donders Institute for Brain, Cognition, and Behaviour, The Netherlands; 3 University Medical Center Utrecht, The Netherlands; 4 De Hoogstraat Rehabilitation, The Netherlands; 5 Karakter, Child and Adolescent Psychiatry, The Netherlands

**Keywords:** autism spectrum disorders, interventions—psychosocial/behavioral, lived experience, peer support, systematic review

## Abstract

The neurodiversity approach recognizes autism as a natural variation of human experience, emphasizing unique strengths while acknowledging social and behavioral challenges that may affect quality of life. Peer support, based on shared experiences and mutual understanding, has shown benefits in mental health care, yet its impact for autistic individuals remains underexplored. This review is of peer-support programs for individuals with autism, focusing on impact, facilitators, and barriers. A systematic literature review was conducted using Cochrane Library, Web of Science, PubMed, Embase, PsycINFO, and Sociological Abstracts. Studies involving peer-support programs for autistic individuals aged 12 and older were selected. Fifteen articles described 12 unique peer-support programs with varying goals, such as enhancing personal development. Studies reported diverse improvements, including enhanced well-being, self-esteem, and academic performance. Many participants valued connecting with peers in an autism-focused context. Most articles discussed some facilitators and barriers. Peer support shows promising benefits for autistic individuals, fostering empowerment and well-being. However, the methodological limitations of the included studies, such as small sample sizes and lack of control groups, limit the strength of these conclusions. Future research should therefore use more robust research methods and investigate accessibility and potential risks to optimize peer support for this population.

**Lay Abstract:**

**Connecting through peer support: Understanding the impact of peer-support programs on individuals with autism and exploring barriers and facilitators.**

**Aim and Purpose of the Research:** This study aims to explore the impact of peer-support programs for autistic individuals. Peer support is defined as a supportive relationship between people with shared lived experiences. This review examines the impact of these programs on autistic individuals and identifies key challenges and facilitators that may influence outcomes.

**Background:** Autism, characterized by differences in social interaction and behavior, can affect many aspects of daily life, including social and academic functioning, which can lead to a reduced quality of life. While peer support has proven beneficial in general healthcare, its potential for autistic individuals remains underexplored. Peer-support programs may offer mutual understanding and emotional support, making them a promising approach to improving well-being for people with autism.

**Methods:** A systematic review was conducted using multiple databases to identify research articles published up to January 17, 2024. Studies included focused on peer-support programs for autistic individuals aged 12 and older, employing methods such as interviews or questionnaires to assess their impact.

**Results and Importance:** The findings indicate that peer-support programs generally have a positive impact, including improved self-esteem, academic performance, and overall well-being. Participants valued the opportunity to connect with others with similar experiences. Although the findings are promising, most studies were small and low quality, so more research is needed. Future research should also investigate the factors contributing to successful peer support and explore ways to optimize these programs for autistic individuals.

## Introduction

Autistic individuals often have distinct sensory, cognitive, and emotional processing styles compared to non-autistic individuals, which may lead to challenges in communication, social interaction, and daily functioning (American Psychiatric Association, 2013). The global prevalence of autism is estimated at 100 per 10,000 individuals and is rising due to increased community awareness and changes in case definitions (Zeidan et al., 2022). Autism may impair quality of life by causing functional limitations in social and academic domains (Wong & Shorey, 2022). In addition, individuals may face challenges related to prejudice and stigma, which can hinder their ability to establish and maintain friendships with non-autistic people, leading to loneliness, and some may experience difficulties with achieving full independence (Friedman & Rizzolo, 2018; Kwan et al., 2020; Mason et al., 2021; Petrina et al., 2014; Ribeiro et al., 2023). Consequently, individuals with autism may require varying levels of support, from none to substantial (American Psychiatric Association, 2013).

To address these challenges, individuals with autism are often offered interventions within the (bio)medical model, which may include psychological therapies or pharmacological treatment for co-occurrent psychiatric problems such as anxiety, depression, and obsessive-compulsive disorder (Hume et al., 2021; Lai et al., 2019). While these interventions may benefit some individuals, they may overlook autistic individuals’ lived experiences and identities. The neurodiversity approach helped extend this (bio)medical view (Pisciotta, 2024), in which any form of neuropsychological development is seen as part of natural variation and holding equal validity (Dwyer, 2022). Rather than seeking to correct autistic traits, neurodiversity-informed approaches emphasize acceptance, empowerment, and the inclusion of autistic voices in the development of support systems (Pisciotta, 2024). This view urges health care to prioritize incorporating the autistic experience in different interventions.

Within this context, peer support has gained attention as a form of support grounded in lived experience (Solomon, 2004). Originating in the mental health field, peer support evolved both as a grassroots response to unmet needs and as a political movement advocating for self-determination and equality in care (Mead et al., 2001; Stratford et al., 2019). Peer support is support based on understanding another’s situation through shared and lived experience, providing knowledge and understanding not accessible through professional expertise alone (Fortuna et al., 2022; Mead et al., 2001). The foundational principles of peer support, reciprocity, shared identity, and empowerment (Fortuna et al., 2022) resonate strongly with neurodiversity-informed models.

Peer support can provide social and emotional support, as well as practical strategies related to managing everyday life, executive functioning, and future planning; it can also foster a sense of belonging and self-worth grounded in mutual understanding and shared identity (Bertilsdotter Rosqvist, 2019; Chen et al., 2021; Salzer & Shear, 2002; Solomon, 2004; Valderrama, 2023). Because peer support relies on mutual identification, self-reflection, and abstract reasoning (Fortuna et al., 2022; Shalaby & Agyapong, 2020), it may be particularly relevant from adolescence onward, when these cognitive and emotional capacities begin to emerge (Crone & Dahl, 2012; Inhelder & Piaget, 1958). Peer support may take place with or without professional facilitation, both online and in-person and involve trained or untrained peers (Fortuna et al., 2022; Naslund et al., 2016; Shalaby & Agyapong, 2020).

Studies exploring peer support across populations and settings report promising outcomes, such as improvements in clinical recovery within mental illnesses (Smit et al., 2023) and an enhanced empowerment in oncology patients (Ziegler et al., 2022). In spite of these promising results, research on peer support with autism being the shared experience remains scarce. Most existing studies focus on peer-mediated interventions involving neurotypical peers rather than peer-led, mutual support among autistic individuals (Chang & Locke, 2016). Therefore, the aim of this review is to provide an overview of the current state of scientific evidence on peer support among autistic individuals aged 12 and older, in order to explore its potential usefulness in health care. This systematic review addresses the following questions: What is the impact of peer-support programs on individuals with autism, and what are key challenges and facilitators associated with peer support in this population?

### Inclusive language statement

This review acknowledges the varying preferences for language when referring to autism, using both identity-first (autistic person) and person-first (person with autism) language (Buijsman et al., 2023).

## Methods

### Study design and data sources

A systematic review was performed, using the Preferred Reporting Items for Systematic Reviews and Meta-Analyses (PRISMA) Checklist (Page et al., 2021) to report this study (see Supplemental Material, File 1). The protocol of the systematic review was registered on the international prospective register of systematic reviews (PROSPERO, registration number: CRD42024491679).

### Data sources and search strategy

The systematic search of literature took place on January 17, 2024, in the following electronic databases: Cochrane Library, Web of Science, PubMed, Embase, PsycINFO, and Sociological Abstracts. The keywords that formed the basis of the search terms and synonyms were: (1) “autism” and (2) “peer support” (see Supplementary Material, File 2). There were no restrictions for publication date during the search. A librarian with expertise in systematic reviews supported the design of the search strategy. The reference lists of the full-text screened articles were manually screened to identify additional studies.

### Eligibility criteria

Included were studies that (1) were original and published in international peer-reviewed journals; (2) were published in English; (3) focused on individuals between 12 and 99 years old with autism; and (4) developed, used, or evaluated a program involving peer support between individuals with autism. The age cutoff reflects that peer support requires cognitive and emotional capacities, such as self-reflection and abstract reasoning, which typically develop from adolescence onward (Crone & Dahl, 2012; Inhelder & Piaget, 1958). As peer support was defined in this review as support provided by and for autistic individuals, grounded in shared lived experience, studies were excluded that (1) focused on peer support between a person with autism and a neurotypical person and (2) did not report on the impact, barriers, or facilitators concerning the peer support. Also, for the purpose of the present systematic review, gray literature and non-peer-reviewed articles were excluded. Although we acknowledge that many valuable practices take place outside of formal research settings.

### Identification and selection of studies

All articles found were loaded into EndNote X9 (The EndNote Team, 2013) to identify and delete potential duplicates. All duplicates were manually checked before deletion. The results were exported into Rayyan (Ouzzani et al., 2016), and titles and abstracts of all entries were screened by two researchers (M.H.M.V. and M.v.W.). Any disagreement between reviewers about inclusion or exclusion was resolved by discussion. In case of lasting doubt, the articles were included for full-text screening. Next, the remaining articles were full text screened by two researchers (M.H.M.V. and M.v.W.). Any disagreement between reviewers or doubt about definite inclusion or exclusion was resolved by discussion with the research team until consensus was reached.

### Data extraction and synthesis

All included articles were assessed by two researchers (M.H.M.V. and M.v.W.) on the following data: study characteristics (e.g., title and country), participant characteristics (e.g., age and gender), program characteristics (e.g., name of the program, setting), and outcomes (e.g., impact on individual, facilitators, or barriers). In addition, program websites were researched to find supplementary information that was not included in the articles. Furthermore, the risk of bias of all included full-text articles was assessed using the Joanna Briggs Institute (JBI) Critical Appraisal Tool (Joanna Briggs Institute, 2017), which is suitable for the design of a study (qualitative, semi-experimental, or narratives). These checklists contain six to ten items with the response options of yes, no, unclear, and not applicable. The higher the number of questions answered with yes, the lower the risk of bias.

### Community involvement statement

This study is a review that synthesizes existing literature and does not involve the collection or interpretation of new empirical data. Therefore, no autistic individuals, family members, or other community stakeholders were directly involved in the research process. We acknowledge the importance of community involvement in autism research and encourage future studies using new data to actively include autistic individuals and other community members in their design and implementation.

## Results

### Study selection

**Figure 1.**
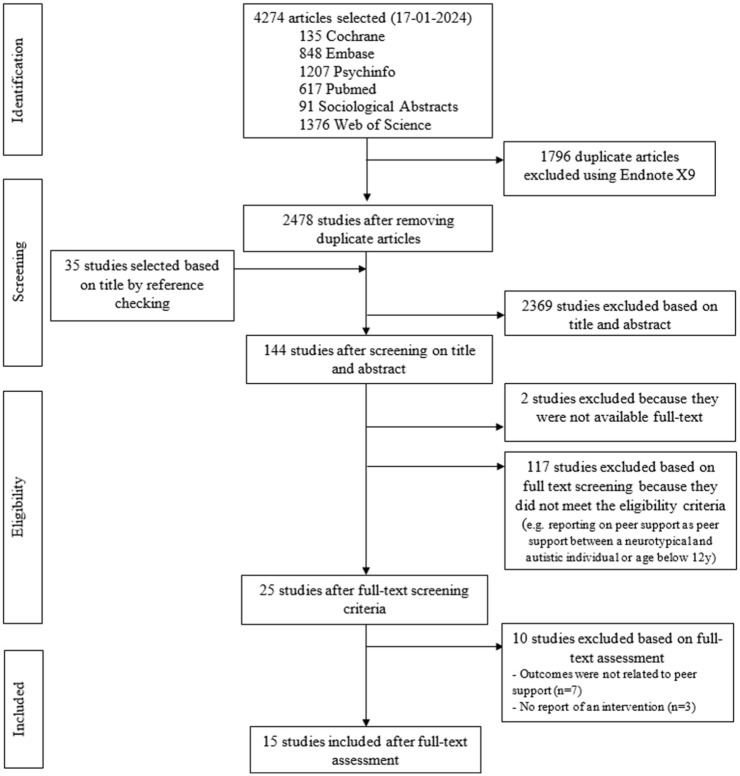
Flow of studies in a systematic review of peer support for individuals with autism, January 2024.

The initial search (dated on January 17, 2024) yielded 4274 articles. After removal of duplicates, the titles and abstracts of 2478 articles were screened, and 142 studies were selected for full-text reading. Title and abstract of 35 additional articles were screened after identification through reference list checking. In total, 15 articles were included in this review (Brownlow et al., 2023; Capozzi et al., 2019; Crane et al., 2021, 2023; Farkas et al., 2020; Hillier et al., 2007; Hotez et al., 2018; Jantz, 2011; MacLeod, 2010; Manett, 2022; McConkey et al., 2021; Shea et al., 2022; Song et al., 2023; Tomfohrde et al., 2022; Weiler et al., 2022) (see Figure 1 for the flowchart).

### Program and study characteristics

**Figure 2.**
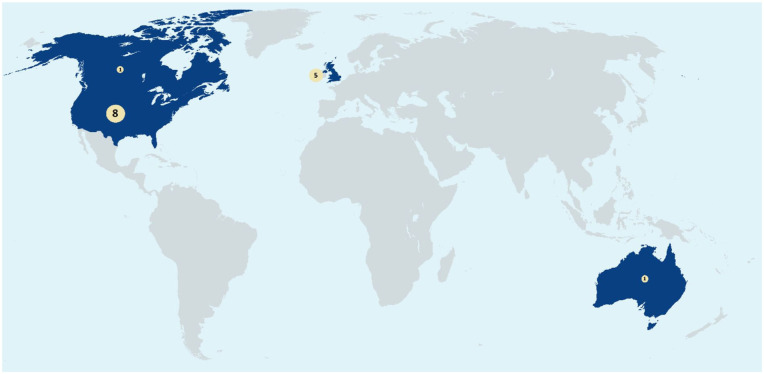
Geographic representation of studies included in this review.

A total of 12 unique peer-support programs were identified in the 15 included studies. The descriptions of the programs are summarized in Table 1. The peer-support programs took place in Western, English-speaking countries (see Figure 2). All included studies were fairly small, with sample sizes ranging from 5 (Tomfohrde et al., 2022) to 54 (McConkey et al., 2021) (see Supplemental Material, File 4). While the specific aims of the programs differed, nearly all included a component of sharing thoughts and experiences with peers in similar situations. More specifically, programs in educational settings aimed to support autistic students in transitioning to and succeeding in higher education, while in mental health care, peer support aimed at fostering self-understanding, emotional well-being, and social inclusion (see Table 1).

**Table 1.** Characteristics of identified peer-support programs.

	Program name	Setting	Goal of the program	Features	Facilitation	Reference
1.	A-SKILLS	- Education - Australia	To provide an autonomous space for autistic students to achieve individual success at university.	- Weekly 90-min meetings - Combined group and individual - Structured and manualized - Combined face to face and digital (videocalls)	- Duration: 10 weeks - Organized by non-autistic professionals - Led by both autistic and non-autistic peers - Facilitating peers received payment and training associated with this role	(Brownlow et al., 2023)
2.	College Bound Academy (CBA)	- Education - USA - Payed program^a^ (California Lutheran University, 2018)	To prepare autistic students who use augmentative and alternative communication to transition to higher education.	- 5-hr meetings^a^ (California Lutheran University, 2018) - Individual - Structured - Face to face	- Duration: 4 days - Organized by non-autistic professionals - Led by both non-autistic professionals and autistic peers	(Capozzi et al., 2019)
3.	Exploring Being Autistic	- Mental health care - UK - Payed program^a^ (Hearst)	To enable learning about autism and to discover if/how it affects someone personally; process emotional response to diagnosis; consider the pros and cons of disclosing that they are autistic; develop strategies to capitalize on the strengths and mitigate the challenges associated with autism; and socialize with peers.	- Weekly 2-hr meetings - Group - Structured and manualized - Digital (videocalls) (Crane et al., 2023) - Face to face (Crane et al., 2021)	- Duration: 10 weeks - Organized and led by one autistic professional^a^	(Crane et al., 2021, 2023)
4.	Autism Work Peer Support Group	- Community (for those actively pursuing employment) - UK	To assist in generating and sharing thoughts, opinions, and concerns, and in making comments on intervention measures in a relaxed, comfortable setting, where the engagement of discussion partners could motivate others and help in expressing their views.	- Fortnightly 90-min meetings - Group - Structured - Combined face to face and digital (forum)	- Duration: at least 12 months - Organized and led by non-autistic professionals (on request of the autistic members)	(Farkas et al., 2020)
5.	Aspirations	- Mental health care - USA	To foster understanding of a range of social and vocational issues, to enhance insight and awareness, and to provide social opportunities.	- Weekly 1-hr meetings - Group - Structured - Face to face	- Duration: 8 weeks + monthly reunions - Organized and led by non-autistic professionals	(Hillier et al., 2007)
6.	The Summer Transition Program	- Education - USA	To help autistic students transition into and succeed in college.	- Daily 5 hr - Group - Face to face	- Duration: 3 weeks - Organized and led by non-autistic professionals	(Hotez et al., 2018)
7.	AS Support Groups	- Mental health care - USA	To provide support and information to the individuals with autism.	- Group - Structured and manualized	- Duration: Varying between 1 and 68 months - Organized and led by both autistic and non-autistic peers	(Jantz, 2011)
8.	AS Portal	- Education - UK	To provide an online network for students on the autism spectrum.	- Group and individual - Digital (forum)	- Duration: Varying between 1 and 5 months - Organized by non-autistic professionals - Led by autistic peers	(MacLeod, 2010)
9.	Social Association for Students with Autism (SASA)	- Education - Canada	To offer a venue for shared group activities and discussions to provide opportunities for students with autism to socialize with each other and to promote social engagement and friendship development.	- Group - Face to face	- Duration: Unknown, open group with new members added as old members leave - Organized and led by non-autistic professionals	(Manett, 2022)
10	Right4U-Adult ASD Service	-Mental health care - UK	To advance personal development and social inclusion and to provide more formal social skills training.	- Fortnightly 2-hr meetings - Group - Face to face	- Duration: 6–12 months - Organized and led by non-autistic professionals	(McConkey et al., 2021)
11	Community Autism Peer Specialist (CAPS)	- Mental health care - USA	To promote independent living, participation, and social relationships of autistic youth and adults.	- Weekly multiple hours meetings - Individual - Structured and manualized - Combined face to face and digital (videocalls)	- Duration: 3 months - Organized and led by autistic peers - Mentoring peers received 75 hr of training associated with this role	(Shea et al., 2022; Song et al., 2023)
12	The Autism Mentorship Program (AMP)	- Education - USA	To provide a buffer against the isolation, emotional difficulties, and social challenges that youth with autism may encounter in adolescence.	- Weekly 1-hr meetings - Group and individual - Manualized - Face to face	- Duration: 12 weeks - Organized by non-autistic professionals - Led by autistic peers - All peers received 2 hr of training associated with this role	(Tomfohrde et al., 2022; Weiler et al., 2022)

*Note.* ASD = autistic spectrum disorder.

a Information found online, not in the article itself.

### Outcomes

All studies reported that peer support had positive effects on the peers *receiving* support, but outcomes and designs varied between studies (see Table 2 and Supplemental Material, File 5). Throughout these varying research designs, being able to connect with like-minded others and a community of autistic individuals was found to be beneficial in most articles (Brownlow et al., 2023; Capozzi et al., 2019; Crane et al., 2021, 2023; Hillier et al., 2007; Manett, 2022; McConkey et al., 2021; Tomfohrde et al., 2022). Furthermore, peer support was found to enhance well-being, empowerment, and acceptance among individuals, with some studies assessing this using interviews (Crane et al., 2021; MacLeod, 2010), while another used both self-developed and validated questionnaires in a pre-test/post-test design (Weiler et al., 2022). Improvements in social skills were found within a validated questionnaire (Song et al., 2023) and with focus groups (McConkey et al., 2021). Furthermore, peer support also led to an increased sense of social connection, with participants noting better communication with both family members and neurotypical peers (Tomfohrde et al., 2022). In addition, participants reported the added benefit of making friends during the program (Hillier et al., 2007).

Six programs did not make a distinction between peers offering and receiving support (Farkas et al., 2020; Hillier et al., 2007; Jantz, 2011; MacLeod, 2010; Manett, 2022; McConkey et al., 2021), as opposed to other programs with peer-support *receivers* and *providers* (Brownlow et al., 2023; Capozzi et al., 2019; Crane et al., 2021, 2023; Hotez et al., 2018; Shea et al., 2022; Song et al., 2023; Tomfohrde et al., 2022; Weiler et al., 2022). Only two studies, both focusing on the Autism Mentorship Program, explored the perspectives of peer-support *providers* in addition to the experiences of the peer-support *receivers* (Tomfohrde et al., 2022; Weiler et al., 2022). In addition, four other studies included experiences of peer-support *providers* in their results, even though this was not the primary focus (Capozzi et al., 2019; Hotez et al., 2018; Manett, 2022; Shea et al., 2022). Across these studies, peer-support *providers* reported in interviews feelings of empowerment and pride in their role (Capozzi et al., 2019; Hotez et al., 2018; Tomfohrde et al., 2022) as well as in validated questionnaires (Weiler et al., 2022). Improved leadership skills were also reported within these questionnaires (Weiler et al., 2022), with similar findings emerging from focus groups (Hotez et al., 2018). Finally, one study reported on the effects of a training on being a mentor; peers *providing* support reported that they were better able to build relationships with their peer, and the training helped them prepare for being a mentor (Tomfohrde et al., 2022).

**Table 2.** Overview of the study characteristics, including results in terms of impact of the program on participants.

	Program name	Research question or study aim	Design and analyses	Data collected	Participants of the program (receiver)	Relevant findings^a^	Reference
1.	A-SKILLS	1. What are participants’ attitudes to, and beliefs about, the support provided by the A-Skills program? 2. What specific areas of learning did A-Skills participants identify as being of most/least benefit?	Design: Action research Analysis: Mixed quantitative and qualitative Follow-up: Not reported	- Semi-structured interviews - Engagement metrics, evaluation data - Student reflections *Participant rating scales*	- *N* = 38 - Mean age = not specified - Severity of ASD = Not specified, but all attend higher education	The program achieved a feeling of community and support; student reflections showed increased confidence in facilitators and improved study management in receivers.	(Brownlow et al., 2023)
2.	College Bound Academy (CBA)	1. This essay presents personal narratives by three nonspeaking autistic college students who mentored 12 non-speaking autistic teenagers and young adults in the College Bound Academy (CBA)	Design: Case study (using narratives) Analysis: Qualitative Follow-up: Not reported	- Personal narratives	- *N* = Not specified - Mean age = not specified - Severity of ASD = Not specified, but all are nonverbal college students	The three narratives report similar experiences with peer support in navigating challenges, more autistic visibility, and a community to help people with autism feel better understood and appreciated.	(Capozzi et al., 2019)
3a	Exploring Being Autistic	1. To identify any benefits of the program for participants 2. To make the program more acceptable to participants in the future	Design: Longitudinal Analysis: Qualitative Follow-up: 6 months after completing the program	- Interviews	- *N* = 17 - Mean age = 44 - Severity of ASD = Not specified, but all diagnosed at adult age	Participants reported to feel less alone due to their diversity-driven cohesion. Furthermore, their outlook on the autism diagnosis improved, resulting in learning and talking about autism and related challenges, which helped them in their day-to-day life.	(Crane et al., 2021)
3b		1. To identify benefits/challenges of the online version of Exploring Being Autistic? 2. To identify the unique challenges/opportunities of the online delivery	Design: Longitudinal Analysis: Qualitative Follow-up: 6-8 months after completing the program	- Interviews - Questionnaires *Expectations and motivation*	- *N* = 16 - Mean age = 49 - Severity of ASD = Not specified, but all diagnosed at adult age	Participants found diversity-driven cohesion and developed a positive and practical outlook on themselves and the autism diagnosis. Outcomes of the in-person and online versions were similar.	(Crane et al., 2023)
4.	Autism Work Peer Support Group	1. To increase resilience in autistic jobseekers by co-developing a subjectively meaningful and purposeful peer-support group at a Job Centre Plus 2. To demonstrate the implementation of Community-Based Participatory Research (CBPR) as a method to enhance engagement and resilience in autistic adults seeking employment through engaging with the autistic community and utilizing their views and opinions in a research design process	Design: Action research and descriptive (post-test) Analysis: Qualitative Follow-up: 12 months after the start of the program	- Questionnaires *CBPR Implementation-Quality Items* *Resilience items*	- *N* = 24 - Mean age = not specified - Severity of ASD = Not specified, but all diagnosed at adult age	Active participation in the accessible AWPSG sessions for autistic job seekers led to significant personal and professional benefits, as it had a positive impact on their self-esteem, social connections, employability skills, and confidence to find and sustain employment.	(Farkas et al., 2020)
5.	Aspirations	1. To review the planning, implementation, and evaluation of Aspirations, an 8-week program designed to foster the development of social and vocational skills for adolescents and young adults on the autism spectrum	Design: Descriptive (pre-test/post-test) Analysis: Mixed quantitative and qualitative Follow-up: Not reported	- Structured observations - Review of the notes taken during staff weekly review meetings - Questionnaires *Self-report measures* *Index of Peer Relations* *Autism Spectrum Quotient Empathy Quotient*	- *N* = 13 - Mean age = 19 - Severity of ASD = Not specified, but all finished higher education	Participants reported they benefited from self-disclosure and they had more respect for other perspectives. Overall they were positive about the program. They made friends and had a more positive attitude toward employers; they developed a more open expression. No significant changes in ASD quotient or peer relations were found, but empathy quotient improved after the program.	(Hillier et al., 2007)
6.	The Summer Transition Program	1. To research the feasibility of a participatory approach wherein autistic college students play a leadership role in the program design and implementation of a summer transition program 2. To identify if participation is associated with enhanced self-advocacy skills, enhanced academic self-efficacy, and/or reduced self-reported ASD symptoms?	Design: Action research and descriptive Analysis: Mixed quantitative and qualitative Follow-up: 6 months after completing the program	- Focus groups - Semi-structured interview - Questionnaires *Academic Self-Efficacy Survey* *Autism Awareness Survey* *Disability Identity and Opportunities Scale* *SRS-A*	- *N* = 10 - Mean age = 19 - Severity of ASD = Not specified, but all attend higher education	Participants developed knowledge of autism, could name strengths associated with autism, and reported less autistic features. They found the mentors helpful and reported no preference for a mentor with or without autism. Participants felt better able to decide whether or not to disclose their autism diagnosis. No differences were found in disability identity or academic self-efficacy.	(Hotez et al., 2018)
7.	AS Support Groups	1. To identify the experiences, gains, and challenges of participation 2. To identify the needs of those not participating 3. To identify reasons for prematurely quitting	Design: Mixed-methods Analysis: Mixed quantitative and qualitative Follow-up: Not reported	- Interviews - Questionnaires *Autism-Spectrum Quotient* *UCLA Loneliness Scale* *Self-developed scale based on How Groups Work scale*	- *N* = 35 - Mean age = not specified - Severity of ASD = Not specified	Participants in a group scored higher on the AQ than the before-intervention group. Participants perceived support groups as beneficial for providing structure, social skills and interaction, and for receiving information and advice.	(Jantz, 2011)
8.	AS Portal	1. To determine the success, functioning, and user-friendliness of the portal 2. To identify if the portal fulfills the need	Design: Action research and descriptive design Analysis: Qualitative Follow-up: Not reported	- Statistics of portal use - Evaluations - Feedback - Discourse analysis - Questionnaire *Participant evaluation sheets*	- *N* = 7 - Mean age = Not specified - Severity of ASD = Not specified but all attend higher education	The portal was difficult to use, and the participation of subjects was the highest in discussions that were initiated by the participants themselves. The content of discussions indicated that students felt safe to share personal experiences, and actively sought and gave advice.	(MacLeod, 2010)
9.	Social Association for Students with Autism (SASA)	1. To describe the background, formation, and facilitation of the SASA	Design: Descriptive design Analysis: Qualitative Follow-up: Not reported	- Interviews	- *N* = Not specified - Mean age = Not specified - Severity of ASD = Not specified but all attend higher education	The program fostered enjoyment in social outings, and offered school support and advice. Members felt that their social skills improved as a result of participating. Members positively reinforced each other’s behaviors by engaging in conversation and activities. Members demonstrating problematic behaviors in a group were corrected by others in a natural way.	(Manett, 2022)
10	Right4U-Adult ASD service	1. To describe the characteristics, needs, and aspirations of people with autism referred to the service since its inception in 2016 2. To identify the impact of the service as perceived by the service users, their relatives, and project personnel 3. To obtain stake-holders’ perceptions and how it could be strengthened	Design: Descriptive design Analysis: Mixed quantitative and qualitative Follow-up: None	- Interviews - Focus groups - Questionnaire *Self-completion questionnaire concerning reaction to the service*	- *N* = 54 - Mean age = 21 - Severity of ASD = Not specified	The program gave participants reasons to get out of the house, taught them to become more confident, and created opportunities to socialize. Participants reported increased self-esteem and confidence, improved social skills, better understanding of themselves, and better stress management as a result of the program.	(McConkey et al., 2021)
11 a	Community Autism Peer Specialist (CAPS)	1. To examine areas of feasibility of CAPS aimed at enhancing self-identified goals for community outcomes among autistic adolescents and adults	Design: Descriptive (post-test) Analysis: Quantitative Follow-up: 3 months after the start of the program	- Questionnaires *Self-report on service utilization* *Working Alliance Inventory* *Satisfaction*	- *N* = 29^b^ - Mean age = 20 - Severity of ASD = Not specified	Participants were highly engaged and satisfied with the program, with 90% of participants and 80% of peer providers reporting overall good satisfaction. Most participants agreed that CAPS peer specialists believed in their autonomy, growth, and responsibility.	(Shea et al., 2022)
11b		1. To assess the preliminary effectiveness of the CAPS program.	Design: Descriptive (pre-test/post-test) Analysis: Quantitative Follow-up: 3 months after the start of the program	- Questionnaires *Social Responsiveness Scale-2* *Modified version of Camberwell Engagement in other services* *Overall Quality of Life*	- *N* = 23^b^ - Mean age = 21 - Severity of ASD = Not specified	The program improved social functioning through better communication and understanding. Participants showed reduced impairments in social functioning, cognition, communication, motivation, and repetitive behaviors at the 3-month follow-up. They also had fewer unmet needs and felt better supported in overall wellness. Quality of life did not change over time.	(Song et al., 2023)
12a	The Autism Mentorship program (AMP)	1. To examine the perceptions of AMP participants regarding their experiences with the program	Design: Descriptive Analysis: Qualitative Follow-up: Not reported	- Focus groups	- *N* = 5 - Mean age = 15 - Severity of ASD = Not specified	The program increased social connectedness and academic performance, satisfied mentors and families, and led to suggestions for community engagement. Parents noted that academic performance increased.	(Tomfohrde et al., 2022)
12b		1. To determine social validity, including uptake, program satisfaction, and mentoring relationship quality 2. To assess changes in well-being, self-concept, and social-emotional and behavioral outcomes	Design: Descriptive (pre-test/post-test) Analysis: Quantitative Follow-up: Not reported	- Questionnaires *- Participant well-being and program questionnaire* *- Strength of Relationship scales* *- Piers–Harris Self-Concept Scale 2* *- The Achenbach* *System of Empirically Based Assessment*	- *N* = 7 - Mean age = Not specified - Severity of ASD = Not specified	AMP benefited both mentees and mentors, improving quality of life, social skills, behavioral regulation, and mental health, with all participants reporting satisfaction and meaningful participation. Mentees felt more comfortable socially, and experienced improved well-being and self-concept.	(Weiler et al., 2022)

*Note.* ASD = autistic spectrum disorder.

a See Supplemental Material, File 5 for an extensive overview.

b All participants who participated in Song et al. (2023) also participated in Shea et al. (2022).

### Barriers and facilitators

Almost all studies reported on barriers or facilitators affecting either the specific program or peer support in general (see Table 3). Facilitators were, for example, active encouragement toward members to be themselves and a flexible format to enhance accessibility such as individual choices in sessions or hybrid formats (Brownlow et al., 2023; Crane et al., 2023; Manett, 2022). Furthermore, the involvement of autistic experts-by-experience during the development, implementation, and evaluation of the peer-support programs was described as being essential for the success of the program (Brownlow et al., 2023; Capozzi et al., 2019; Crane et al., 2021; Farkas et al., 2020; Hotez et al., 2018; Tomfohrde et al., 2022) (see Table 3). Reported barriers for peer support were logistical impediments such as scheduling issues, lack of meeting structure, or bureaucratic processes in the health care system (Hotez et al., 2018; Jantz, 2011; McConkey et al., 2021). It was noted that some facilitators were also reported as barriers and vice versa.

**Table 3.** Overview of reported facilitators and barriers.

	Program name	Facilitators	Barriers	Reference
1	A-SKILLS	- Online availability: Revisit information in an online environment and possibilities for individualization of the program - Frequent sessions: Weekly sessions resulted in a feeling of cohesion - Autistic peer: Peer delivered, sharing autistic experiences	- Not personalized: No individualization of the in-person delivered part of the program - Perceived lack of safety: Group sessions not exclusive to autistic students - Online delivery: Less relatedness than in-person delivered	(Brownlow et al., 2023)
2	College Bound Academy (CBA)	N/S	N/S	(Capozzi et al., 2019)
3	Exploring Being Autistic	- Peer group diversity: Members were at different stages of their diagnostic process, which created a sense of belonging, which was considered especially important - Autistic peer: Peer delivered	- No follow-up: Lack of follow-up support may result in a feeling of uncertainty and feeling directionless	(Crane et al., 2021)
	Exploring Being Autistic	- Online delivery: Reduced cognitive load, enhanced accessibility, and facilitated meaningful connections - Not personalized: Flexibility in format (in-person, online, or hybrid) made the support accessible to a broad range of autistic people	- Technical issues: Resulting in less time for the peer support itself - Online delivery: Online socialization was perceived as difficult with the loss of some human aspects, such as recognizing emotions on-screen - No facilitator: Lack of scaffolding when divided into smaller groups	(Crane et al., 2023)
4	Autism Work Peer Support Group	- Autistic peer: Involvement in the development resulted in a relaxed and safe environment, and a meaningful community group - Sense of connectedness: Openly sharing and learning from peers	N/S	(Farkas et al., 2020)
5	Aspirations	N/S	N/S	(Hillier et al., 2007)
6	The Summer Transition Program	- Collaborated development: peer participation in the process of developing enhanced program satisfaction yielded a sense of empowerment among peers and drew from strengths associated with autism that are likely to be helpful, such as heightened attention to detail and honesty	- Logistical concerns: Challenges in scheduling meetings	(Hotez et al., 2018)
7	AS Support Groups	- Structured meetings: By including time limits, choosing topics in advance, setting an agenda and providing homework, meetings were more effective - Group composition: Preference for members of similar ages, equal gender ratio, or similarities in employment or personal situations - Fixed group: Minimal turnover in participants resulted in friendships		(Jantz, 2011)
8	AS Portal	- Not personalized: Individualization to learning styles and networking culture - Online availability: A portal with a space for discussion - Open access: Less intimidating than having to ask permission	N/S	(MacLeod, 2010)
9	Social Association for Students with Autism (SASA)	- Not personalized: a flexible meeting structure encouraged members to “be themselves” and allowed for engaging in a variety of ways that were often unconventional	N/S	(Manett, 2022)
10	Right4U-Adult ASD service	N/S	- Motivation: not all participants were equally motivated, and some did not take the initiative in organizing social events	(McConkey et al., 2021)
11	Community Autism Peer Specialist (CAPS)	N/S	N/S	(Shea et al., 2022)
	Community Autism Peer Specialist (CAPS)	- Not personalized: Peers identified and addressed the needs among one another and adjusted the program subsequently - Facilitating mutual understanding: Empowered participants to enhance their social functioning - Translating emotions: Peer-facilitators identified emotions and learned their peer’s alternative strategies for responding to them	N/S	(Song et al., 2023)
12	The Autism Mentorship Program (AMP)	- Autistic peer: Peer delivered, sharing autistic experiences - Staff support: When feeling challenged, peers could ask staff support for suggestions concerning the peer support	- Feeling unprepared: Peers felt unsure how to deepen and how to handle large group activities, due to a lack of conversational skills	(Tomfohrde et al., 2022)
	The Autism Mentorship Program (AMP)	- Staff support: Active and regular engagement of all stakeholders offered continues input and improvement of the program	N/S	(Weiler et al., 2022)

*Note.* N/S = not specified; ASD = autistic spectrum disorder.

### Risk of bias

A risk-of-bias assessment was performed on each of the 15 included full-text articles, using the JBI Critical Appraisal Tool (Joanna Briggs Institute, 2017) suitable for each study design. As for these studies, the quality generally met the JBI standards; however, none of the studies complied with all the criteria (see Supplemental Material, File 6). For example, none of them included a control group, and only five studies reported a follow-up period with a minimum of 3 months (Shea et al., 2022) and a maximum of 12 months after the start of the program (Farkas et al., 2020). Overall, the qualitative studies met more of the JBI criteria than the quasi-experimental studies.

## Discussion

This review aimed to provide an overview of the existing literature on peer-support programs for autistic individuals. Importantly, gray literature and informal peer-support initiatives were not included in our review, as they often fall outside formal evaluation frameworks, even though they may constitute a substantial part of peer-support initiatives all over the world. Fifteen studies were identified, which described 12 unique peer-support programs for individuals with autism aged 12 and older. Although structural differences were found among the programs applying peer support, and studies examining the same program used various evaluation methods, all studies described an overall positive impact of peer support provided by and to individuals with autism.

In all studies, participants expressed that as a result of the peer-support program, they felt better understood and empowered, and that they experienced a sense of belonging by being part of a group of people sharing similar challenges and experiences (Capozzi et al., 2019; Crane et al., 2021; Hillier et al., 2007; Hotez et al., 2018; MacLeod, 2010). These results align with the theoretical foundation of peer support, which emphasizes acceptance, understanding, and socialization (Valderrama, 2023). Furthermore, our results suggest that participants of the peer-support programs not only learned coping strategies for potential challenges associated with autism but also gained self-understanding and confidence (Hotez et al., 2018; Weiler et al., 2022). Considering these results in light of what is known about peer support, the opportunity to discuss and reflect with a peer may be especially valuable in gaining a personal understanding of what autism means on a personal level (Salzer & Shear, 2002). This may lead to a more favorable perception of autistic traits (Fortuna et al., 2022), which was seen in two of the included mixed-methods studies, with results highlighting increased confidence and self-advocacy skills (Hotez et al., 2018; McConkey et al., 2021). Other included studies reported improvements in social skills, communication, and friendships (MacLeod, 2010; McConkey et al., 2021; Song et al., 2023; Tomfohrde et al., 2022). Other positive outcomes consisted of academic improvements (Tomfohrde et al., 2022), decreased needs for mental health services (Song et al., 2023), and increased well-being (Weiler et al., 2022). Furthermore, *providing* peer support was reported to enhance empowerment, leadership skills, and self-confidence (Capozzi et al., 2019; Hotez et al., 2018; Manett, 2022; Shea et al., 2022; Tomfohrde et al., 2022; Weiler et al., 2022).

True to the definition and concept of peer support we used (Fortuna et al., 2022), we chose to focus on peer support with autistic peers only and excluded peer support delivered by neurotypical peers (Bertilsdotter Rosqvist, 2019; Chang & Locke, 2016; Morris et al., 2024). Whereas non-autistic individuals may share experiences with the school or work context, they lack the experience of living with autism (Bertilsdotter Rosqvist, 2019). Our results from the present review showed that peer support between autistic peers was highly valued among participants (Brownlow et al., 2023; Crane et al., 2021, 2023; Farkas et al., 2020; Tomfohrde et al., 2022). This may be because of similar lived experiences (Fortuna et al., 2022), fewer stigmatized viewpoints toward autistic spectrum disorder (ASD) by peer “providers” (Gillespie-Lynch et al., 2017), more empathy toward each other (Komeda et al., 2015), more mutual understanding (Milton et al., 2022), and a general preference among autistic individuals to communicate with other autistic peers (Morrison et al., 2020; Sledge et al., 2011). This aligns with relational learning theories, such as Coactive Vicarious Learning, which propose that learning is most effective when knowledge is co-constructed through mutual reflection and shared interpretation of lived experiences, rather than through one-way instruction or observation (Myers, 2018).

Various barriers and facilitators were identified, with findings showing that what functions as a facilitator in one context may serve as a barrier in another, both within and across studies. For example, an online peer-support format was experienced in different studies as both a facilitator and as a barrier (Brownlow et al., 2023; Crane et al., 2023; MacLeod, 2010; Manett, 2022), and this was the same for diversity of the peer group (Crane et al., 2021; Jantz, 2011). Reported factors that specifically facilitated peer support included customizing the program to meet individual needs, such as offering flexibility in format (Crane et al., 2023), and adapting the program to accommodate various learning preferences (MacLeod, 2010). Preparing peer-support providers by training and education, and in-the-moment support by program staff for peer providers, was reported as helpful and facilitating (Song et al., 2023; Tomfohrde et al., 2022). Examples of factors exclusively reported as barriers were a lack of feeling related to other participants and scheduling issues (Brownlow et al., 2023; Hotez et al., 2018).

Studies investigating peer support typically include individuals interested in participating. It is valuable to examine the perspectives of individuals who are unwilling to participate or who have discontinued a peer-support program prematurely to get more understanding of barriers. Only one study explored this; reasons for refusal to participate included discomfort with sharing sensitive topics, fear of judgment, fear of being mistreatment, concerns about upsetting others, sensory overload, and social anxiety (Jantz, 2011). The same study also identified a lack of direction during meetings, large group size, and difficulty being around other adults living atypical lives as reasons for quitting prematurely (Jantz, 2011). One other important finding was that none of the included studies reported harmful or adverse effects of the interventions. Only one study provided information about challenges, showing conversational difficulties within the mentoring (Tomfohrde et al., 2022).

### Strengths and limitations

To our knowledge, this is the first systematic review on peer support in autism, offering unique insights. A strength is the rigorous methodology; we conducted a comprehensive search in numerous databases, and we screened over 5% of the full-text articles to identify whether peer support involved neurotypical or autistic peers. This approach reduced the risk of missing studies and enhanced the reliability of this review. Limitations include the quality of the included studies, none meeting all appraisal criteria (see Supplemental Material, File 6), complicating comparisons between studies, programs, or outcomes and increasing bias risk. This aligns with challenges in novel psychological interventions, often marked by inconsistent methods and limited validated evaluation tools (Chacón-Moscoso & Sanduvete-Chaves, 2017). Outcome measures sometimes diverged from research aims, and publication bias favoring positive results cannot be ruled out. In addition, our results only included studies from Western, English-speaking countries, limiting generalizability and insights into peer support across other cultural and health care contexts. Furthermore, our findings may not apply to individuals under 12 or those with significant functional challenges.

### Future research

Our review identified promising peer-support programs for individuals with autism, but methodological limitations hinder firm conclusions. None of the included studies used a control group, and follow-up was often absent or short-term. Future research should focus on more robust research methods, ideally using randomized controlled designs with adequate sample sizes, validated outcome measures, and sufficient follow-up. Mixed methods may enrich the understanding of peer-support interventions, especially when these methods are conceptually grounded and focused on outcomes like participation, empowerment, and well-being. Including perspectives of individuals who declined or discontinued participation could enhance accessibility and relevance. Potential adverse effects, such as the burden of hearing about suicidal thoughts or distress in the peer-support relationship, should be further researched.

Involving autistic individuals throughout the research process is essential. While some programs engaged peers in design, most did not, even though peer support fundamentally relies on lived experience (Hotez et al., 2018). Co-design and participatory approaches are likely to improve the fit and sustainability of interventions (Cairns & Nicholls, 2018). Furthermore, future research should examine the development and impact of peer support in more diverse settings, as current academic literature focuses mainly on education and health care. Future research should also examine how factors like age, training, mode of delivery, social relationships, cultural norms, and organizational frameworks shape their effectiveness and acceptability. Although peer support may offer a cost-effective alternative to professional care, only one study reported on this (McConkey et al., 2021). Further economic evaluations are needed.

Importantly, our review excluded gray literature and informal peer-support initiatives, which often fall outside scientific research despite potentially representing a substantial part of peer-support practices. Future research should therefore include environmental scans or mapping studies to capture this broader landscape, ensuring a more comprehensive understanding of peer support for autistic individuals beyond peer-reviewed evidence. Finally, future research should also provide transparent descriptions of the content and processes of peer-support programs to enable replication and the identification of key elements that underpin effectiveness. A deeper understanding of these contextual and structural influences is critical to designing accessible, responsive, and impactful peer support.

### Conclusion

This review aimed to explore the impact of peer-support programs for individuals with autism. Overall, all peer-support programs impacted positively on autistic participants, fostering empowerment and overall well-being, despite structural differences between programs. We found that peer-support methods, styles, and goals vary widely, and its key elements are not yet clearly defined. Despite these variations, peer support remains a promising intervention that can be implemented in diverse settings and formats. The methodological limitations in the included studies highlight the need for more robust methodologies to evaluate the effects of peer support for autistic individuals and examine barriers, facilitators, and accessibility of these programs to facilitate uptake and implementation.

## Supplemental Material

sj-docx-1-aut-10.1177_13623613251374971 – Supplemental material for Impact of peer-support programs for individuals with autism: A systematic review Supplemental material, sj-docx-1-aut-10.1177_13623613251374971 for Impact of peer-support programs for individuals with autism: A systematic review by Monica HM Verkooijen, Marjolijn Ketelaar, Max van Woerden, Wouter G Staal, Indira Tendolkar and Janneke R Zinkstok in Autism

sj-docx-2-aut-10.1177_13623613251374971 – Supplemental material for Impact of peer-support programs for individuals with autism: A systematic review Supplemental material, sj-docx-2-aut-10.1177_13623613251374971 for Impact of peer-support programs for individuals with autism: A systematic review by Monica HM Verkooijen, Marjolijn Ketelaar, Max van Woerden, Wouter G Staal, Indira Tendolkar and Janneke R Zinkstok in Autism
