# Supplementary material for: Impact of peer-support programs for individuals with autism: A systematic review
Source: Autism. 2025 Sep 16;30(2):300–15. doi: 10.1177/13623613251374971 (PMC12804424; doi:10.1177/13623613251374971)
Supplement: sj-docx-2-aut-10.1177_13623613251374971 – Supplemental material for Impact of peer-support programs for individuals with autism: A systematic review [file sj-docx-2-aut-10.1177_13623613251374971.docx]

**Supplemental Material, file 1: PRISMA checklist**

| **Section and Topic** | **Item #** | **Checklist item** | **Location where item is reported** |
| --- | --- | --- | --- |
| **TITLE** | | |  |
| Title | 1 | Identify the report as a systematic review. | Title page |
| **ABSTRACT** | | |  |
| Abstract | 2 | See the PRISMA 2020 for Abstracts checklist. | Abstract |
| **INTRODUCTION** | | |  |
| Rationale | 3 | Describe the rationale for the review in the context of existing knowledge. | Pg. 1-2 |
| Objectives | 4 | Provide an explicit statement of the objective(s) or question(s) the review addresses. | Pg. 2 |
| **METHODS** | | |  |
| Eligibility criteria | 5 | Specify the inclusion and exclusion criteria for the review and how studies were grouped for the syntheses. | Pg. 2-3 |
| Information sources | 6 | Specify all databases, registers, websites, organizations, reference lists and other sources searched or consulted to identify studies. Specify the date when each source was last searched or consulted. | Pg. 3 |
| Search strategy | 7 | Present the full search strategies for all databases, registers and websites, including any filters and limits used. | Supplemental Material, file 2 |
| Selection process | 8 | Specify the methods used to decide whether a study met the inclusion criteria of the review, including how many reviewers screened each record and each report retrieved, whether they worked independently, and if applicable, details of automation tools used in the process. | Pg. 3 |
| Data collection process | 9 | Specify the methods used to collect data from reports, including how many reviewers collected data from each report, whether they worked independently, any processes for obtaining or confirming data from study investigators, and if applicable, details of automation tools used in the process. | Pg. 3-4 |
| Data items | 10a | List and define all outcomes for which data were sought. Specify whether all results that were compatible with each outcome domain in each study were sought (e.g. for all measures, time points, analyses), and if not, the methods used to decide which results to collect. | - |
|  | 10b | List and define all other variables for which data were sought (e.g. participant and intervention characteristics, funding sources). Describe any assumptions made about any missing or unclear information. | - |
| Study risk of bias assessment | 11 | Specify the methods used to assess risk of bias in the included studies, including details of the tool(s) used, how many reviewers assessed each study and whether they worked independently, and if applicable, details of automation tools used in the process. | Pg. 4 and Supplemental Material, file 6 |
| Effect measures | 12 | Specify for each outcome the effect measure(s) (e.g. risk ratio, mean difference) used in the synthesis or presentation of results. | - |
| Synthesis methods | 13a | Describe the processes used to decide which studies were eligible for each synthesis (e.g. tabulating the study intervention characteristics and comparing against the planned groups for each synthesis (item #5)). | Pg. 3-4, and Supplemental Material, file 3 |
|  | 13b | Describe any methods required to prepare the data for presentation or synthesis, such as handling of missing summary statistics, or data conversions. | Pg. 3-4 |
|  | 13c | Describe any methods used to tabulate or visually display results of individual studies and syntheses. | - |
|  | 13d | Describe any methods used to synthesize results and provide a rationale for the choice(s). If meta-analysis was performed, describe the model(s), method(s) to identify the presence and extent of statistical heterogeneity, and software package(s) used. | - |
|  | 13e | Describe any methods used to explore possible causes of heterogeneity among study results (e.g. subgroup analysis, meta-regression). | - |
|  | 13f | Describe any sensitivity analyses conducted to assess robustness of the synthesized results. | - |
| Reporting bias assessment | 14 | Describe any methods used to assess risk of bias due to missing results in a synthesis (arising from reporting biases). | Pg. 3-4 |
| Certainty assessment | 15 | Describe any methods used to assess certainty (or confidence) in the body of evidence for an outcome. | Pg. 3-4 |
| **RESULTS** | | |  |
| Study selection | 16a | Describe the results of the search and selection process, from the number of records identified in the search to the number of studies included in the review, ideally using a flow diagram. | Pg. 4-5 |
|  | 16b | Cite studies that might appear to meet the inclusion criteria, but which were excluded, and explain why they were excluded. | - |
| Study characteristics | 17 | Cite each included study and present its characteristics. | Pg. 4-18 |
| Risk of bias in studies | 18 | Present assessments of risk of bias for each included study. | Pg. 16 and Supplemental Material, file 6 |
| Results of individual studies | 19 | For all outcomes, present, for each study: (a) summary statistics for each group (where appropriate) and (b) an effect estimate and its precision (e.g. confidence/credible interval), ideally using structured tables or plots. | Pg. 11-15 |
| Results of syntheses | 20a | For each synthesis, briefly summarize the characteristics and risk of bias among contributing studies. | Pg. 11-15 and Supplemental Material, file 6 |
|  | 20b | Present results of all statistical syntheses conducted. If meta-analysis was done, present for each the summary estimate and its precision (e.g. confidence/credible interval) and measures of statistical heterogeneity. If comparing groups, describe the direction of the effect. | - |
|  | 20c | Present results of all investigations of possible causes of heterogeneity among study results. | - |
|  | 20d | Present results of all sensitivity analyses conducted to assess the robustness of the synthesized results. | - |
| Reporting biases | 21 | Present assessments of risk of bias due to missing results (arising from reporting biases) for each synthesis assessed. | - |
| Certainty of evidence | 22 | Present assessments of certainty (or confidence) in the body of evidence for each outcome assessed. | - |
| **DISCUSSION** | | |  |
| Discussion | 23a | Provide a general interpretation of the results in the context of other evidence. | Pg. 19-22 |
|  | 23b | Discuss any limitations of the evidence included in the review. | Pg. 21 |
|  | 23c | Discuss any limitations of the review processes used. | Pg. 21-22 |
|  | 23d | Discuss implications of the results for practice, policy, and future research. | Pg. 19-22 |
| **OTHER INFORMATION** | | |  |
| Registration and protocol | 24a | Provide registration information for the review, including register name and registration number, or state that the review was not registered. | Pg. 3 |
|  | 24b | Indicate where the review protocol can be accessed, or state that a protocol was not prepared. | Pg. 3 |
|  | 24c | Describe and explain any amendments to information provided at registration or in the protocol. | - |
| Support | 25 | Describe sources of financial or non-financial support for the review, and the role of the funders or sponsors in the review. | Title Page |
| Competing interests | 26 | Declare any competing interests of review authors. | Title Page |
| Availability of data, code and other materials | 27 | Report which of the following are publicly available and where they can be found: template data collection forms; data extracted from included studies; data used for all analyses; analytic code; any other materials used in the review. | Title Page |

**Supplemental Material, file 2: Search strategy for each database**

|  | **PubMed** |
| --- | --- |
| **#1** | "Autism Spectrum Disorder"[Mesh] OR Autism[tiab] OR Autist*[tiab] OR ASD[tiab] OR "Asperger Syndrome"[tiab] OR "Asperger's Syndrome"[tiab] OR "Aspergers Syndrome"[tiab] OR "Asperger Disease*"[tiab] OR "Asperger's Disease*"[tiab] OR "Asperger Disorder*"[tiab] OR "Asperger's Disorder*"[tiab] OR "Aspergers Disorder*"[tiab] OR "Kanner's Syndrome"[tiab] OR "Kanner Syndrome"[tiab] OR "Pervasive developmental disorder*"[tiab] OR "Pervasive development disorder*"[tiab] OR PDD[tiab] OR PDDNOS[tiab] OR "PDD-NOS"[tiab] |
| **#2** | (Peer group [MeSH] AND social support [MeSH]) OR (("Peer Group*"[tiab] OR "Peer Relation*"[tiab]) AND (Care[tiab] OR Support[tiab])) OR Tutor*[tiab] OR "Peer Support"[tiab] OR "Peers Support"[tiab] OR P2PS[tiab] OR "Peer Coaching"[tiab] OR "Peer Counseling"[tiab] OR "Peer Counselling"[tiab] OR Buddy[tiab] OR Mentor*[tiab] OR "Peer Deliver*"[tiab] OR "Peer meeting*"[tiab] OR "Expert by experience"[tiab] OR "Support Group*"[tiab] OR "Peer mediated intervention*"[tiab] OR PMI [tiab] OR "Peer-mediated intervention*"[tiab] OR "Lived experience*"[tiab] |
| **#3** | 1 and 2 |
| **#4** | Limit 3 to (Dutch or English) |
|  | **Web of Science** |
| **#1** | TS=(Autism OR Autist* OR ASD OR "Asperger Syndrome" OR "Asperger's Syndrome" OR "Aspergers Syndrome" OR "Asperger Disease*" OR "Asperger's Disease*" OR "Asperger Disorder*" OR "Asperger's Disorder*" OR "Aspergers Disorder*" OR "Kanner's Syndrome" OR "Kanner Syndrome" OR "Pervasive developmental disorder*" OR "Pervasive development disorder*" OR PDD OR PDDNOS OR "PDD-NOS") |
| **#2** | TS=((("Peer Group*" OR "Peer Relation*") AND (Care OR Support)) OR Tutor* OR "Peer Support" OR "Peers Support" OR P2PS OR "Peer Coaching" OR "Peers Coaching" OR "Peer Counseling" OR "Peers Counseling" OR "Peer Counselling" OR "Peers Counselling" OR Buddy OR Mentor* OR "Peer Deliver*" OR "Peer meeting*" OR "Expert by experience" OR "Support Group*" OR "Peer mediated intervention*" OR PMI OR "Peer-mediated intervention*" OR "Lived experience*" ) |
| **#3** | #1 AND #2 AND LANGUAGE: (English OR Dutch) |
|  | **Psych info** |
| **#1** | Exp autism spectrum disorders/ OR Autism.ti,ab,id. OR Autist*.ti,ab,id. OR ASD.ti,ab,id. OR "Asperger Syndrome".ti,ab,id. OR "Asperger's Syndrome".ti,ab,id. OR "Aspergers Syndrome".ti,ab,id. OR "Asperger Disease*".ti,ab,id. OR "Asperger's Disease*".ti,ab,id. OR "Asperger Disorder*".ti,ab,id. OR "Asperger's Disorder*".ti,ab,id. OR "Aspergers Disorder*".ti,ab,id. OR "Kanner's Syndrome".ti,ab,id. OR "Kanner Syndrome".ti,ab,id. OR "Pervasive developmental disorder*".ti,ab,id. OR "Pervasive development disorder*".ti,ab,id. OR PDD.ti,ab,id. OR PDDNOS.ti,ab,id. OR "PDD-NOS".ti,ab,id. |
| **#2** | Exp Peer Counseling/ OR Exp Peer Tutoring/ OR (("Peer Group*".ti,ab,id. OR "Peer Relation*".ti,ab,id.) AND (Care.ti,ab,id. OR Support.ti,ab,id.)) OR Tutor*.ti,ab,id. OR "Peer Support".ti,ab,id. OR "Peers Support".ti,ab,id. OR P2PS.ti,ab,id. OR "Peer Coaching".ti,ab,id. OR "Peer Counseling".ti,ab,id. OR "Peer Counselling".ti,ab,id. OR Buddy.ti,ab,id. OR Mentor*.ti,ab,id. OR "Peer Deliver*".ti,ab,id. OR "Peer meeting*".ti,ab,id. OR "Expert by experience".ti,ab,id. OR "Support Group*".ti,ab,id. OR "Peer mediated intervention*".ti,ab,id. OR PMI .ti,ab,id. OR "Peer-mediated intervention*".ti,ab,id. OR "Lived experience*".ti,ab,id. |
| **#3** | 1 and 2 |
| **#4** | Limit 3 to (Dutch or English) |
|  | **Embase** |
| **#1** | Exp autism/ OR Autism.ti,ab,kf. OR Autist*.ti,ab,kf. OR ASD.ti,ab,kf. OR "Asperger Syndrome".ti,ab,kf. OR "Asperger's Syndrome".ti,ab,kf. OR "Aspergers Syndrome".ti,ab,kf. OR "Asperger Disease*".ti,ab,kf. OR "Asperger's Disease*".ti,ab,kf. OR "Asperger Disorder*".ti,ab,kf. OR "Asperger's Disorder*".ti,ab,kf. OR "Aspergers Disorder*".ti,ab,kf. OR "Kanner's Syndrome".ti,ab,kf. OR "Kanner Syndrome".ti,ab,kf. OR "Pervasive developmental disorder*".ti,ab,kf. OR "Pervasive development disorder*".ti,ab,kf. OR PDD.ti,ab,kf. OR PDDNOS.ti,ab,kf. OR "PDD-NOS".ti,ab,kf. |
| **#2** | Exp Peer group/ AND exp social support/ OR (("Peer Group*".ti,ab,kf. OR "Peer Relation*".ti,ab,kf.) AND (Care.ti,ab,kf. OR Support.ti,ab,kf.)) OR Tutor*.ti,ab,kf. OR "Peer Support".ti,ab,kf. OR "Peers Support".ti,ab,kf. OR P2PS.ti,ab,kf. OR "Peer Coaching".ti,ab,kf. OR "Peer Counseling".ti,ab,kf. OR "Peer Counselling".ti,ab,kf. OR Buddy.ti,ab,kf. OR Mentor*.ti,ab,kf. OR "Peer Deliver*".ti,ab,kf. OR "Peer meeting*".ti,ab,kf. OR "Expert by experience".ti,ab,kf. OR "Support Group*".ti,ab,kf. OR "Peer mediated intervention*".ti,ab,kf. OR PMI .ti,ab,kf. OR "Peer-mediated intervention*".ti,ab,kf. OR "Lived experience*".ti,ab,kf. |
| **#3** | 1 and 2 |
| **#4** | Limit 3 to (Dutch or English) |
|  | **Cochrane** |
| **#1** | [mh "Autism Spectrum Disorder"] OR Autism:ti,ab,kw OR Autist*:ti,ab,kw OR ASD:ti,ab,kw OR (Asperger NEXT Syndrome):ti,ab,kw OR (Asperger's NEXT Syndrome):ti,ab,kw OR (Aspergers NEXT Syndrome):ti,ab,kw OR (Asperger NEXT Disease*):ti,ab,kw OR (Asperger's NEXT Disease*):ti,ab,kw OR (Asperger NEXT Disorder*):ti,ab,kw OR (Asperger's NEXT Disorder*):ti,ab,kw OR (Aspergers NEXT Disorder*):ti,ab,kw OR (Kanner's NEXT Syndrome):ti,ab,kw OR (Kanner NEXT Syndrome):ti,ab,kw OR (Pervasive NEXT developmental NEXT disorder*):ti,ab,kw OR (Pervasive NEXT development NEXT disorder*):ti,ab,kw OR PDD:ti,ab,kw OR PDDNOS:ti,ab,kw OR (PDD-NOS):ti,ab,kw |
| **#2** | ([mh "Peer group"] AND [mh "social support"]) OR (((Peer NEXT Group*):ti,ab,kw OR (Peer NEXT Relation*):ti,ab,kw) AND (Care:ti,ab,kw OR Support:ti,ab,kw)) OR Tutor*:ti,ab,kw OR (Peer NEXT Support):ti,ab,kw OR (Peers NEXT Support):ti,ab,kw OR P2PS:ti,ab,kw OR (Peer NEXT Coaching):ti,ab,kw OR (Peer NEXT Counseling):ti,ab,kw OR (Peer NEXT Counselling):ti,ab,kw OR Buddy:ti,ab,kw OR Mentor*:ti,ab,kw OR (Peer NEXT Deliver*):ti,ab,kw OR (Peer NEXT meeting*):ti,ab,kw OR (Expert NEXT by NEXT experience):ti,ab,kw OR (Support NEXT Group*):ti,ab,kw OR (Peer NEXT mediated NEXT intervention*):ti,ab,kw OR PMI:ti,ab,kw OR (Peer-mediated NEXT intervention*):ti,ab,kw OR (Lived NEXT experience*):ti,ab,kw |
| **#3** | 1 and 2 |
| **#4** | Limit 3 to (Dutch or English) |
|  | **Sociological Abstracts** |
| **#1** | MAINSUBJECT.EXACT.EXPLODE("Autism")  OR  ti("Autism spectrum disorder*" OR Autist* OR ASD OR "Asperger syndrome" OR "Asperger's Syndrome" OR "Aspergers Syndrome" OR "Asperger disease" OR "Asperger's disease" OR "Aspergers disease" OR "Aspergers Disorder" OR "Asperger Disorder" OR "Asperger's Disorder" OR "Kanner Syndrome" OR "Kanner's Syndrome" OR "Pervasive Developmental Disorder" OR "Pervasive Development Disorder" OR PDD OR PDDNOS OR "PDD-NOS")  OR  ab("Autism spectrum disorder*" OR Autist* OR ASD OR "Asperger syndrome" OR "Asperger's Syndrome" OR "Aspergers Syndrome" OR "Asperger disease" OR "Asperger's disease" OR "Aspergers disease" OR "Aspergers Disorder" OR "Asperger Disorder" OR "Asperger's Disorder" OR "Kanner Syndrome" OR "Kanner's Syndrome" OR "Pervasive Developmental Disorder" OR "Pervasive Development Disorder" OR PDD OR PDDNOS OR "PDD-NOS")  OR  if("Autism spectrum disorder*" OR Autist* OR ASD OR "Asperger syndrome" OR "Asperger's Syndrome" OR "Aspergers Syndrome" OR "Asperger disease" OR "Asperger's disease" OR "Aspergers disease" OR "Aspergers Disorder" OR "Asperger Disorder" OR "Asperger's Disorder" OR "Kanner Syndrome" OR "Kanner's Syndrome" OR "Pervasive Developmental Disorder" OR "Pervasive Development Disorder" OR PDD OR PDDNOS OR "PDD-NOS") |
| **#2** | MAINSUBJECT.EXACT("Social support") OR TITLE(Care OR Support) OR ABSTRACT(Care OR Support) OR IF(Care OR Support)  AND  MAINSUBJECT.EXACT.EXPLODE("Peer Groups") OR TITLE("Peer Group*" OR "Peer Relation") OR ABSTRACT("Peer Group*" OR "Peer Relation") OR IF("Peer Group*" OR "Peer Relation")  OR  TITLE(Tutor* OR "Peer Support" OR "Peers Support" OR P2PS OR "Peer Coaching" OR "Peer Counseling" OR Buddy OR Mentor* OR "Peer Delver*" OR "Peer Meeting" OR "Expert by Experience" OR "Support Group*" OR "Peer mediated intervention*" OR PMI OR "Peer-mediated intervention*" OR "Lived experience*" ) OR ABSTRACT(Tutor* OR "Peer Support" OR "Peers Support" OR P2PS OR "Peer Coaching" OR "Peer Counseling" OR Buddy OR Mentor* OR "Peer Delver*" OR "Peer Meeting" OR "Expert by Experience" OR "Support Group*" OR "Peer mediated intervention*" OR PMI OR "Peer-mediated intervention*" OR "Lived experience*" ) OR IF(Tutor* OR "Peer Support" OR "Peers Support" OR P2PS OR "Peer Coaching" OR "Peer Counseling" OR Buddy OR Mentor* OR "Peer Delver*" OR "Peer Meeting" OR "Expert by Experience" OR "Support Group*" OR "Peer mediated intervention*" OR PMI OR "Peer-mediated intervention*" OR "Lived experience*" ) |
| **#3** | 1 and 2 |
| **#4** | Limit 3 to (Dutch and English) |

**Supplemental Material, file 3: Data extraction procedure**

This procedure can be used to assess the articles which are included based full text screening. It was drawn up to assess the included articles full text, based on six components: 1) general information article 2) Intervention 3) Participants 4) Peers 5) Outcome 6) Other.

| **Component 1: General information article** | |
| --- | --- |
| Author | Open question |
| Title | Open question |
| Year | Open question |
| Country | Open question |
| Study design | Open question |
| Research question and aim | Open question |
| Does the research question/aim contain peer to peer support? | Yes / No |
| **Component 2: Intervention** | |
| Describe the intervention short, including the name | Open question |
| What is the setting of the intervention? | Education  Mental health care  Social media  In community  Other |
| What is the duration of the intervention | <3 months  3-6 months  6 months – 1 year  1-2 year  >2 years  Unknown  Other |
| What type of P2PS is it | Face to face  Digital  Other |
| Is the P2PS in group or individual setting | Group setting  Individual  Combined group/individual |
| Is the P2PS structured? | Yes / No |
| Do the participants receive any training beforehand? | Yes: Describe this short  Maybe: Describe this short  No |
| **Component 3: Participants** | |
| How many participants joined the intervention (receiving support)? | Open question |
| Note their descriptive data | Gender  Age  Ethnicity  Comorbidity  Severity of the disability? |
| Is there a control group? | Yes: Note their descriptive data   - Gender - Age - Ethnicity - Comorbidity - Severity of the disability   No: Continue to component 4 |
| **Component 4: Peers** | |
| Are the peers who give support, also the ones who receive support? | Yes: Continue to Component 5  No |
| How many participants give support? | Open question |
| Note their descriptive data (of the peer giving support | Gender  Age  Ethnicity  Comorbidity  Severity of the disability? |
| **Component 5: Outcome** | |
| Note a summary of the outcome | Open question |
| What is the primary outcome? | Open question |
| Is a questionnaire or instrument used? | Yes (Name them) / No |
| Is the effect on the peer receiving support described? | Yes (Describe this) / No |
| Is the effect on the peer giving support described? | Yes (Describe this) / No |
| What is the setting of the intervention? | Open question |
| Are barriers towards P2PS noted in the article? | Yes (What are they) / No |
| Are facilitators towards P2PS noted in the article? | Yes (What are they) / No |
| **Component 6: Critical appraisal** | |
| What critical appraisal tool did you use, how many ‘yes’ answers were given? | Open question |
| **Component 7: Supplemental notes** | |
| Do you have any supplemental notes or comments? | Open question |

**Supplemental Material, file 4: Overview of participant characteristics**

|  | **Name of the P2PS program** | **Number of participants** | **Gender** | **Age (mean, SD, range)** | **Ethnicity** | **Comorbidity** | **Severity of ASD** | **Information about peers giving support** | **Reference** |
| --- | --- | --- | --- | --- | --- | --- | --- | --- | --- |
| 1. | A-SKILLS | 38 | Not specified | Not specified | Not specified | Not specified | Not specified, but all attend higher education | Not specified | (Brownlow et al., 2023) |
| 2. | College Bound Academy (CBA) | Not specified | Not specified | Not specified | Not specified | Not specified | Not specified, but all are nonverbal college students | 3 peers support providers, not further specified | (Capozzi et al., 2019) |
| 3. | Exploring Being Autistic | 16 | 11 female 5 male | Mean = 44.43  SD = N/S  Range = 18-71 | Not specified | Not specified | Not specified, but diagnosed at adult age | Not specified | (Crane et al., 2021) |
|  | Exploring Being Autistic | 17 | 10 female 6 male 1 other | Mean = 49.2  SD = 9.62  Range = 30-79 | Not specified | Not specified | Not specified, but diagnosed at adult age | Not specified | (Crane et al., 2023) |
| 4. | Autism Work Peer Support Group | 24 | 7 female 17 male | Mean = N/S  SD = N/S  Range = 18-46 | Not specified | Not specified | Not specified, but all finished higher education | Same as participants | (Farkas et al., 2020) |
| 5. | Aspirations | 13 | 2 female 11 male | Mean=19 SD = N/S  Range = 18-23 | Not specified | Not specified | Not specified | Same as participants | (Hillier et al., 2007) |
| 6. | The summer transition program | 10 | 2 female 8 male | Mean = 18.8  SD = 1.58  Range = 17-22 | 6 white,  2 black,  2 Asian | Not specified | Not specified, but all attending higher education | Not specified | (Hotez et al., 2018) |
| 7. | AS support groups | 35 | 11 female 24 male | Mean = N/S  SD = N/S  Range = 25-75 | Not specified | Not specified | Not specified | Same as participants | (Jantz, 2011) |
| 8. | AS portal | 7 | Not specified | Not specified | Not specified | Not specified | Not specified, but all attending higher education | Same as participants | (MacLeod, 2010) |
| 9. | Social Association for Students with Autism (SASA) | Not specified | Not specified | Not specified | Not specified | Not specified | Not specified, but all attend higher education | Same as participants | (Manett, 2022) |
| 10. | Right4U-Adult ASD service | 54 | 15 female  39 male | Mean = 21  SD = N/S  Range = 17-51 | 54 Irish/ British white | 12 with mental health issues (e.g. anxiety, low mood, OCD or PTSD) | Not specified | Same as participants | (McConkey et al., 2021) |
| 11. | Community Autism Peer Specialist (CAPS) | 29 ^A^ | 6 female 23 male | Mean = 19.7  SD = 6.1  Range = 14-41 | 12 black, 13 white,  4 Hispanic | 8 ADHD, 6 depression, 9 anxiety, 2 PTSD, 2 bipolar disorder | Not specified | N=6 (4 female, 2 male)  Age: Mean=29.7; SD=28; Range=21-46  Ethnicity: 3 white, 1 black, 1 native pacific islander, 1 other  Comorbidity: 2 ADHD, 2 depression, 2 anxiety | (Shea et al., 2022) |
|  | Community Autism Peer Specialist (CAPS) | 23 ^A^ | 4 female 19 male | Mean = 20.57 SD = 6.47  Range = 14-41 | 10 black,  9 white,  4 Hispanic | 4 ADHD, 3 depression, 5 anxiety, 2 PTSS, 1 ID | Not specified | Not specified, but all were >18 years | (Song et al., 2023) |
| 12. | The Autism Mentorship Program (AMP) | 5 | 5 male | Mean = 15.4  SD = 0.73  Range = 15-17 | 3 Hispanic,  2 non-Hispanic | 4 ADHD, 3 anxiety, 1 social anxiety, 1 PTSD | Not specified | N=6 (5 female, 1 male)  Age: Mean=28.64; SD=5.4; Range=21-34,  Ethnicity: 6 non-Hispanic | (Tomfohrde et al., 2022) |
|  | The Autism Mentorship Program (AMP) | 7 | 7 male | Mean = N/S  SD = N/S  Range = 14-16 | 2 White, 3 Hispanic, 2 have >1 ethnicity | 5 ADHD, 4 anxiety, 3 other various conditions (e.g. PTSD) | Not specified | N=7 (2 female, 5 male)  Age: Mean/SD N/S, Range=19-33  Ethnicity: 6 white, 1 unknown  Comorbidity: 4 anxiety, 3 ADHD, 2 depression | (Weiler et al., 2022) |

^A^ All participants who participated in Song (Song et al., 2023) also participated in Shea (Shea et al., 2022)

**Supplemental Material, file 5: Overview of impact on participants**

| 10 | **Name of the P2PS program** | **Design and analyses** | **Qualitative findings concerning the effect on the peer receiving support** | **Effect on peer receiving support** | **Effect on peer providing support** | **Conclusion of the authors** | **Reference** |
| --- | --- | --- | --- | --- | --- | --- | --- |
| 1. | A-SKILLS | Design: Action research  Analysis: Mixed quantitative and qualitative  Follow-up: Not reported | There are no themes specified.  Peer support offers the possibility to connect with like-minded others and the sense of a community with an autistic context. | Student reflections:  Goals were met: 3.58  Better able to manage studies: 3.06  Facilitators were supportive: 4.67  Confidence in Facilitators: 4.25  Overall benefit: 4.50  *Score 1 to 5* | Not reported | Peer support programs, such as A-skills, have a role to play to engaging students with ASD and assisting them in navigating their way through the university experience. | (Brownlow et al., 2023)^A^ |
| 2. | College Bound Academy (CBA) | Design: Case study (using narratives)  Analysis: Qualitative  Follow-up: Not reported | There are no themes specified.  Participants felt more understood and appreciated to having a role model who has gone through similar experiences  Peer support offers to learn about navigating challenges and victories, supporting peers by knowledge gained from living through similar challenges. Increasing autistic visibility and representation, giving a voice to individuals with ASD and building a community and hope in a good future. | Not applicable | They felt pride to contribute to better preparing individuals with ASD for higher education. | The personal narratives demonstrate the possible benefits of including peers with ASD in the support of individuals with ASD | (Capozzi et al., 2019) |
| 3a | Exploring Being Autistic | Design: Longitudinal  Analysis: Qualitative  Follow-up: 6 months after completing the program | Three themes are specified  1. Appreciation of the autistic nature of the Program  2. Unity in Diversity  3. Developing a Positive and Practical Outlook on Autism  Participants felt not alone.  Peer support improved their outlook on their diagnosis of and the strengths of ASD, resulting in more acceptance, empowerment, and self-awareness. This helped participants in their day-to-day lives. | Not applicable | Not reported | This was a successful initial evaluation of this P2PS program, the autistic-led nature of the program was seen as a particularly positive aspect of the program. | (Crane et al., 2021) |
| 3b | Exploring Being Autistic | Design: Longitudinal  Analysis: Qualitative  Follow-up: 6-8 months after completing the program | There are no themes specified.  Participants appreciated the autistic-led nature of the program, found unity in the diversity of the group, and developed a positive and practical outlook following their participation | Not applicable | Not reported | This program’s online versions evaluation yielded similar results to the evaluation of the in-person version (Crane et al., 2021). While it identified positive and negative aspects of the online delivery. | (Crane et al., 2023) |
| 4. | Autism Work Peer Support Group (AWPSG) | Design: Action research and descriptive (post-test)  Analysis: Qualitative  Follow-up: 12 months after start of the program | There are no themes specified.  Participation in the AWPSG had a positive impact on the participants’ self-esteem, social connections, employability skills and confidence to find and sustain employment.  Participation in the program led to significant personal and professional benefits as they formed friendships and social connections, which in some cases have directly led to new employment opportunities | Not applicable | Not reported | This study supports the efficacy of this P2PS program and provide a framework for providing employment focused P2PS to this population. | (Farkas et al., 2020) |
| 5. | Aspirations | Design: Descriptive (pre-test/post-test)  Analysis: Mixed quantitative and qualitative  Follow-up: Not reported | Observational data indicated an improved recognition and respect for other perspectives. Furthermore, participants developed a positive attitude towards the program, and they appreciated and gained from the discussions about experiences and situations.  Cohesiveness developed, the majority seemed comfortable in the group, as demonstrated by their increased self-disclosure. As sessions continued there was increased self-disclosure and more contributions were made than in the beginning.  Participants noted that they made friends with other in the group and had an increased effort to interact with others socially. They reported more positive attitudes toward gaining employment. Participants benefited to interact with other individuals who are going through similar experiences | Index of peer relations:  No significant improvement was found (z=4.454, p =0.146)  Autism Spectrum Quotient:  No significant decrease was found (z=0.105, p= 0.916)  Modified Empathy Quotient:  Significant decrease was found (z =2.520, p =0.012). | Not reported | The experience of being accepted into a group, meeting others with ASD, and having the opportunity to discuss challenging issues seemed to have a positive impact on participants | (Hillier et al., 2007)^A^ |
| 6. | The summer transition program | Design: Action research and descriptive  Analysis: Mixed quantitative and qualitative  Follow-up: 6 month after completing the program | Three themes are specified  1. Defining self-advocacy: students’ definitions of self-advocacy did not change substantively  2. Strengths in disability: an increase in ASD knowledge and to name a strength in their disability was found. Furthermore, a decrease in self-reported ASD symptoms was observed.  3. Factors deciding disclosing at college  (at post-test): There was an increase in participants that evaluated circumstantial factors to decide whether to disclose their disability.  No changes in disability identity or academic self-efficacy were observed  Learned general and college specific skills.  At the 6-month follow-up. Participants described using their new skills. They found the mentors helpful and had no preference to a mentor with or without ASD. | Self-reported ASD traits: Significant decrease was found (z = -2.14, p =0.03).  ASD knowledge: Significant decrease was found (z =-2.21, p =0.03).  Disability pride:  No significant difference was found (z =-0.71, p =0.47)  Feelings of exclusion: No significant difference was found (z =-0.34, p =0.73)  Social model orientation: No significant difference was found (z =-0.50, p =0.62)  Medical model orientation: No significant difference was found (z =-0.33, p =0.74)  Self-efficacy: No significant difference was found (z =-0.07, p =0.94) | Autistic and non-autistic mentor felt empowered by sharing their perspectives and knowledge and it developed their leadership skills. | This research suggests that participation in a brief but intensive summer transition program may help prepare autistic college students to self-advocate and engage with diverse peers in college contexts. | (Hotez et al., 2018)^A^ |
| 7. | AS support groups | Design: Descriptive design  Analysis: Mixed quantitative and qualitative  Follow-up: Not reported | Four themes were indicated for reasons for attending:  1. Social skills and interaction: Participants felt a sense of belonging and learned how people interact  2. Information and advice: Participants learned about each other’s ASD traits  3. Structure: Serving as structured time in a week  4. They were encouraged to  attend by someone else  Participants perceived support groups as  beneficial for providing social skills and interaction, information and advice and for structure | Positive correlation between the AQ and UCLA Loneliness Scale score (r = .334, p ≤ .05).  Negative correlation between the UCLA Loneliness Scale  score and the number of social engagements (r = –.398, p ≤ .05).  There was no association between the number of months in an AS support group and the UCLA Loneliness Scale  score (r = -.039, ns) or between connections to psychiatric support (meaning either a therapist or a psychiatrist or both) and the UCLA Loneliness Scale score (r = .06, ns) | - | The program suggested that P2PS was helpful to acquire social skills, social interaction and information about ASD. | (Jantz, 2011) ^A^ |
| 8. | AS portal | Design: Action research and descriptive design  Analysis: Qualitative  Follow-up: Not reported | The content of discussions in this study indicated that students did feel safe to share personal experiences of their lives as students on the autism spectrum. Within the student-initiated messages, individuals reflected on their situations and actively sought and gave advice.  The number of responses were higher in discussion that were initiated by participants and had a higher number of participants contributing to them.  The portal has the potential to reduce feelings of isolation for new students, by providing the supportive and informal student network, but the space itself was difficult to use and visually poor. All participants deemed the content quite relevant. | Not applicable | Not reported | The portal was successful in fostering P2PS. Participants were mostly responsive to discussions initiated by fellow peers. Moreover, the most productive discussions were related to ASD. However, the space itself was difficult to use and visually poor. | (MacLeod, 2010) ^A^ |
| 9. | Social Association for Students with Autism (SASA) | Design: Descriptive design  Analysis: Qualitative  Follow-up: Not reported | There are no themes specified.  Members positively reinforced each other’s behaviors by engaging in conversation topics, activities, and ways of interacting that were mutually enjoyable. When members demonstrated behaviors that were problematic in the context of the group, other members gave more naturalistic feedback through negative reactions and/or requests to change the behavior.  Peer support offered the opportunity to enjoy activities and outings, satisfying a previously unmet desire for socialization, and accessing advice and support around challenges at school. | Not applicable | The benefits identified above reflect the mechanisms of mutual aid discussed earlier. As university students with ASD, group members shared characteristics, goals, and challenges in common. | SASA was created to address challenges with adjusting to and integrating within the university and it is suggested that it enhances members’ experience and wellbeing. | (Manett, 2022) |
| 10. | Right4U-Adult ASD service | Design: Descriptive design  Analysis: Mixed quantitative and qualitative  Follow-up: None | Four themes were identified:  1. Out of the House: the program gave participants reasons to get out of the house. 2. Socializing: it created opportunities to socialize  3. Experiences: it provided participants with new experiences in terms of new places to visit or novel activities.  4. Learning: users learned to become more confident and social skills  Participants learned from the program, resulting in an increased self-esteem and confidence, worrying less, better cope with stress, improved social skills and better understanding themselves. | Self-reported changes: The changes most frequently reported were spending more time out of the house, greater independence and more confidence. Service users were less inclined to report changes in terms of getting or holding down a job or being more capable of taking further training. | Not reported | This study adds to the evidence that low level, community-based interventions can reduce social isolation, improve social networks and community participation in adults with ASD. | (McConkey et al., 2021) ^A^ |
| 11 | Community Autism Peer Specialist (CAPS) | Design: Descriptive (post-test)  Analysis: Quantitative  Follow-up: 3 months after start of the program | Not applicable | 90% participants reported overall satisfaction with the program, and participants were generally highly engaged in the services. | 80% of the peer provider reported overall satisfaction with the program. All peer specialists were  sensitive to the cultural or ethnic background of the people they worked with; encouraged them to take responsibility for how they lived their life; believed that they could grow and change; and were willing to see them as often as they felt it were necessary | This study suggest that CAPS can be feasible in the community setting. With positive peer relationships and high satisfaction. | (Shea et al., 2022) |
|  | Community Autism Peer Specialist (CAPS) | Design: Descriptive (pre-test/post-test)  Analysis: Quantitative  Follow-up: 3 months after start of the program | Not applicable | Social Responsiveness Scale-2:  Significant decreases were observed in four out of five domains: social cognition (z=-2.93, p=0.003), social communication (z=-2.77, p=0.006), social motivation (z=-2.42, p=0.016), and restricted interests and repetitive behavior (z=-30.07, p=0.002)  Modified Camberwell Assessment of Need: Statistic significant decrease in needs were found  (9.35 vs. 5.57; z=-2.89, p=0.004).  Lehman’s Quality of Life: No significant differences over time were found:  (M=4.27±1.55 vs. M=4.14±1.61, z=-0.44, p=0.662). | Not reported | This study shows that the CAPS program could benefit individuals with ASD with a wide range of needs. | (Song et al., 2023) |
| 12a | The Autism Mentorship Program (AMP) | Design: Descriptive  Analysis: Qualitative  Follow-up: Not reported | Four themes were identified:  1. Perceived program benefits: all stakeholders experienced more social connectedness and better academic performance.  2. Support for the mentoring relationship: mentors were overall satisfied with their training.  3. Family engagement and support: families were overall content with their engagement.  4. Suggestions for future programming: more variety of games to be played and more engagement in the community  Participants perceived a number of benefits in the area of social connectedness, and an increase in academic performance was  noted by their parents. | Not applicable | P2PS providers reported their own hesitation and struggles in conversations and interactions with P2PS receivers. They felt a tendency for surface-level conversations.  Mentors also noted an increase in their sense of social connectedness. Mentors liked having someone to spend time with | This study supports a mentoring-based service for and by individuals with ASD. | (Tomfohrde et al., 2022) |
| 12b | The Autism Mentorship Program (AMP) | Design: Descriptive (pre-test/post-test)  Analysis: Quantitative  Follow-up: Not reported | Not applicable | The majority of mentees (85.7%) reported satisfaction with their participation and the support they received. Mentees rated the quality of their mentoring relationship as 4.00 (5-point scale).  Well-being: A small increase in quality of life (*g*=0.34), life satisfaction (*g*=0.34), enjoyment in life (*g*=0.70), self-satisfaction (*g=*0.46), relationship satisfaction (*g=*0.73) was noted. No change was reported in pride in autistic identity (*g=*0.00) or frequency of negative feelings (*g=*0.00).  Self-concept: An increase in overall self-concept was noted (*g=*0.64)  Social-emotional and behavioral outcomes: An decrease in internalizing problems (g=-0.38) and externalizing problems (g=-0.87) was noted. | The majority of mentors (85.7%) reported satisfaction with their participation. Mentors rated the quality of  their relationship at 3.89 (5-point scale).  Mentors reported  greater confidence, more patience, and better leadership skills as a result of participating in the program, furthermore an increase on well-being outcome measures was found, and a decrease in self-concept measures. | This study suggests that one-to-one mentoring for and by individuals with ASD is an acceptable intervention strategy and can possibly promote positive outcomes. | (Weiler et al., 2022) |

**Supplemental Material, file 6: Critical Appraisal score**

| **Quasi-experimental studies** | **(Brownlow et al., 2023)** | **(Farkas et al., 2020)** | **(Hillier et al., 2007)** | **(Hotez et al., 2018)** | **(McConkey et al., 2021)** | **(Shea et al., 2022)** | **(Song et al., 2023)** | **(Weiler et al., 2022)** |
| --- | --- | --- | --- | --- | --- | --- | --- | --- |
| 1. Is it clear in the study what is the ‘cause’ and what is the ‘effect’ (i.e. there is no confusion about which variable comes first)? | Yes | No | Yes | Yes | Yes | Yes | Yes | Yes |
| 2. Were the participants included in any comparisons similar? | Yes | Not applic-able | Not applic-able | Not applic-able | Yes | Not applic-able | Yes | Yes |
| 3. Were the participants included in any comparisons receiving similar treatment/care, other than the exposure or intervention of interest? | Yes | Not applic-able | Not applic-able | Not applic-able | Yes | Not applic-able | Yes | Yes |
| 4. Was there a control group? | No | No | No | No | No | No | Unclear | Unclear |
| 5. Were there multiple measurements of the outcome both pre and post the intervention /exposure? | No | No | Yes | Yes | Yes | No | Yes | Yes |
| 6. Was follow up complete and if not, were differences between groups in terms of their follow up adequately described and analyzed? | Unclear | Yes | Unclear | Yes | No | Yes | Yes | Unclear |
| 7. Were the outcomes of participants included in any comparisons measured in the same way? | Unclear | Not applic-able | Not applic-able | Not applic-able | Yes | Not applic-able | Yes | Yes |
| 8. Were outcomes measured in a reliable way? | Unclear | Yes | Yes | Yes | Yes | Yes | Yes | Yes |
| 9. Was appropriate statistical analysis used? | Not applic-able | Not applic-able | Yes | Yes | No | Yes | Yes | Yes |

| **Textual evidence - narrative** | **(Capozzi et al., 2019)** | **(MacLeod, 2010)** |
| --- | --- | --- |
| 1. Is the generator of the narrative a credible or appropriate source? | Yes | Yes |
| 2. Is the relationship between the text and its context explained? (where, when, who with, how) | No | No |
| 3. Does the narrative present the events using a logical sequence so the reader or listener can understand how it unfolds? | No | Yes |
| 4. Do you, as reader or listener of the narrative, arrive at similar conclusions to those drawn by the narrator? | Yes | Yes |
| 5. Do the conclusions flow from the narrative account? | Yes | Yes |
| 6. Do you consider this account to be a narrative? | Unclear | No |

| **Qualitative Research** | **(Crane et al., 2021)** | **(Crane et al., 2023)** | **(Jantz, 2011)** | **(Manett, 2022)** | **(Tomfohrde et al., 2022)** |
| --- | --- | --- | --- | --- | --- |
| 1. Is there congruity between the stated philosophical perspective and the research methodology? | Yes | Yes | Yes | Unclear | Yes |
| 2. Is there congruity between the research methodology and the research question or objectives? | Yes | Yes | Yes | Unclear | Yes |
| 3. Is there congruity between the research methodology and the methods used to collect data? | Yes | Yes | Yes | Unclear | Yes |
| 4. Is there congruity between the research methodology and the representation and analysis of data? | Yes | Yes | Yes | Not applic-able | Yes |
| 5. Is there congruity between the research methodology and the interpretation of results? | Yes | Yes | Yes | Unclear | Yes |
| 6. Is there a statement locating the researcher culturally or theoretically? | No | No | No | No | Yes |
| 7. Is the influence of the researcher on the research, and vice- versa, addressed? | No | No | No | No | No |
| 8. Are participants, and their voices, adequately represented? | Yes | Yes | Yes | Yes | Yes |
| 9. Is the research ethical according to current criteria or, for recent studies, and is there evidence of ethical approval by an appropriate body? | Yes | Yes | No | Unclear | Yes |
| 10. Do the conclusions drawn in the research report flow from the analysis, or interpretation, of the data? | Yes | Yes | Yes | Yes | Yes |

References

Brownlow, C., Martin, N., Thompson, D. M., Dowe, A., Abawi, D., Harrison, J., & March, S. (2023). Navigating University: The Design and Evaluation of a Holistic Support Programme for Autistic Students in Higher Education. *Education Sciences*, *13*(5). <https://doi.org/doi:10.3390/educsci13050521>

Capozzi, S., Barmache, D., Cladis, E., Peña, E. V., & Kocur, J. (2019). The Significance of Involving Nonspeaking Autistic Peer Mentors in Educational Programs. *Autism Adulthood*, *1*(3), 170-172. <https://doi.org/doi:10.1089/aut.2019.0006>

Crane, L., Hearst, C., Ashworth, M., & Davies, J. (2023). Evaluating the online delivery of an autistic-led programme to support newly diagnosed or identified autistic adults. *Autism Dev Lang Impair*, *8*, 23969415231189608. <https://doi.org/doi:10.1177/23969415231189608>

Crane, L., Hearst, C., Ashworth, M., Davies, J., & Hill, E. L. (2021). Supporting Newly Identified or Diagnosed Autistic Adults: An Initial Evaluation of an Autistic-Led Programme. *J Autism Dev Disord*, *51*(3), 892-905. <https://doi.org/doi:10.1007/s10803-020-04486-4>

Farkas, T. N., Mendy, J., & Kargas, N. (2020). Enhancing Resilience in Autistic Adults Using Community-based Participatory Research: A Novel HRD Intervention in Employment Service Provision. *Advances in Developing Human Resources*, *22*(4), 370-386. <https://doi.org/doi:10.1177/1523422320946293>

Hillier, A., Fish, T., Cloppert, P., & Beversdorf, D. Q. (2007). Outcomes of a social and vocational skills support group for adolescents and young adults on the autism spectrum. *Focus on Autism and Other Developmental Disabilities*, *22*(2), 107-115. <https://doi.org/doi>:

Hotez, E., Shane-Simpson, C., Obeid, R., DeNigris, D., Siller, M., Costikas, C., Pickens, J., Massa, A., Giannola, M., D'Onofrio, J., & Gillespie-Lynch, K. (2018). Designing a Summer Transition Program for Incoming and Current College Students on the Autism Spectrum: A Participatory Approach. *Front Psychol*, *9*, 46. <https://doi.org/doi:10.3389/fpsyg.2018.00046>

Jantz, K. M. (2011). Support Groups for Adults With Asperger Syndrome. *Focus on Autism and Other Developmental Disabilities*, *26*(2), 119-128. <https://doi.org/doi:10.1177/1088357611406903>

MacLeod, A. (2010). 'Welcome to my first rant!' Report on a participatory pilot project to develop the 'AS portal', an online peer support network for higher education students on the autism spectrum. *Journal of Assistive Technologies*, *4*(1), 14-24. <https://doi.org/doi:10.5042/jat.2010.0041>

Manett, J. (2022). The social association for students with autism: Principles and practices of a social group for university students with ASD. *Social Work with Groups*, *45*(2), 157-171. <https://doi.org/doi>:

McConkey, R., Cassin, M. T., McNaughton, R., & Armstrong, E. (2021). Enhancing the social networks of adults with ASD: a low level community intervention. *Advances in Autism*, *7*(4), 322-334. <https://doi.org/doi:10.1108/aia-07-2020-0043>

Shea, L. L., Wong, M. Y., Song, W., Kaplan, K., Uppal, D., & Salzer, M. S. (2022). Autistic-Delivered Peer Support: A Feasibility Study. *J Autism Dev Disord*, 1-14. <https://doi.org/doi:10.1007/s10803-022-05816-4>

Song, W., Salzer, M. S., Kaplan, K., Wong, M. Y., Uppal, D., & Shea, L. L. (2023). Short Report on Effectiveness of an Autistic-Delivered Peer Support Program: Preliminary Results. *Community Ment Health J*. <https://doi.org/doi:10.1007/s10597-023-01174-2>

Tomfohrde, O., Hudock, R. L., Kremer, K. B., Fatiha, N., & Weiler, L. (2022). Fostering social connectedness among adolescents and adults with autism: A qualitative analysis.DP - Jun 25, 2022. *Psychology in the Schools*, No Pagination Specified. <https://doi.org/doi>:

Weiler, L. M., Goerdt, A. K., Kremer, K. B., Goldberg, E., & Hudock, R. L. (2022). Social Validity and Preliminary Outcomes of a Mentoring Intervention for Adolescents and Adults With Autism. *Focus on Autism and Other Developmental Disabilities*, *37*(4), 215-226. <https://doi.org/doi:10.1177/10883576211073687>
